# Chemotherapy Response as a Predictor of Survival in Post-neoadjuvant Esophagectomy Specimens

**DOI:** 10.7759/cureus.84963

**Published:** 2025-05-28

**Authors:** Hira Yaqub, Sajid Mushtaq, Mudassar Hussain, Hina Maqbool, Safana Sadaf

**Affiliations:** 1 Pathology, Shaukat Khanum Memorial Cancer Hospital and Research Centre, Lahore, PAK

**Keywords:** chemotherapy, esophagectomy, prognosis, response, survival

## Abstract

Background

Esophageal carcinoma is one of the most common cancers worldwide, which is conventionally treated with neoadjuvant chemoradiation and surgery. Presurgical treatment impacts outcomes by various factors.

Objective

This study aims to evaluate the prognostic significance of chemotherapy response in esophagectomy specimens and its association with survival outcomes in a post-neoadjuvant setting.

Methods

One hundred eighteen in-house post-neoadjuvant chemoradiation resection cases for esophageal carcinoma at Shaukat Khanum Memorial Cancer Hospital and Research Centre, Lahore, Pakistan, with a follow-up period of at least six years were included in the study. Clinical T and N stage, current status and sites, and time of disease progression were retrieved from hospital archives. Pathological T and N stage and chemotherapy response scores (CRSs), along with various other factors, were assessed during the histopathological review. Overall survival (OS) and progression-free survival (PFS) were calculated and statistically analyzed by IBM SPSS Statistics for Windows, Version 29 (Released 2021; IBM Corp., Armonk, New York, United States).

Results

In a study of 118 patients, 68 (57.6%) died. The median OS was 65 months, and the five-year survival rate was 59.6%. Prognostic factors identified in the univariate analysis included age, clinical T stage, pathological T stage, pathological N stage, and CRS. ypT0 had a median survival of 72 months, significantly longer than ypT1 (65 months), ypT2 (36 months), and ypT3 (24 months). Similar trends were observed for improving CRSs. Disease progression occurred in 37 patients (31.35%), with a median PFS of 82.93 months. Multivariate analysis revealed that only CRS score 2 was statistically significant in predicting OS outcomes.

Conclusion

The study found a positive correlation between improved CRSs and OS/PFS, as well as between decreased pathological T stage and survival. It highlights the need for grading systems to include downstaged lymph nodes in therapy response assessments.

## Introduction

Squamous cell carcinoma is the most commonly observed esophageal carcinoma worldwide [1]. The second most common type, adenocarcinoma, is less frequently present in the distal third of the esophagus. Unlike other sites in the gastrointestinal tract, where adenocarcinoma is the predominant type, esophageal carcinoma has two subtypes (squamous cell carcinoma and adenocarcinoma) that behave differently. Studies have shown that early-stage squamous cell carcinomas have a worse prognosis than adenocarcinoma [2]. Resection of the invasive tumor, endoscopic resection for very early disease, and esophagectomy for advanced stage are the mainstays of treatment. Neoadjuvant chemoradiation is included in the treatment for extensive disease to improve outcomes [3]. Chemoradiation helps stop micrometastasis and often demotes the tumor stage [4-5]. Even for clinical T4 disease, neoadjuvant chemoradiation improves long-term survival by showing partial or complete response in the resection specimens [4]. A significant relation can be seen between the pretherapy tumor length (gross tumor size) and response. Other factors that fail to predict the response include differentiation of tumor, tumor site, smoking, and alcohol history [6].

Chemoradiation imparts significant changes in the resection specimen itself, imposing further challenges for the pathologist to identify viable tumors. Grossly, the tumor bed can show a scar, ulcer, erythema, mucosal irregularity, or residual tumor. Microscopically, there can be degenerative atypia of cells, therapy-induced nuclear pleomorphism, or cytoplasmic eosinophilia. The tumor bed can have inflammation, calcifications, fibrosis, granulation tissue, atrophy of submucosal glands, intimal proliferation of vessels, foreign body giant cell reaction, acellular mucin pools, and keratin debris. Quantification of the residual viable tumor cells by percentage proves to be an important individual predictor of prognosis [7]. Alternatively, residual cancer volume (number of cassettes involved) can also be used as a means of assessing response and thus predicting prognosis [8]. Different systems have been proposed over time for grading tumor regression.

Therapy-associated changes in the lymph node also have individual prognostic implications. Therapy changes are quantitatively depicted by the presence of a residual tumor and qualitatively by fibrosis, inflammation, and giant cells. Lymph nodes with any of the therapy changes described for the tumor bed and lacking viable tumor cells should be counted for therapy response [3]. While a lot has been studied regarding the neoadjuvant chemotherapy response in esophageal cancer worldwide, data on the Pakistani population is significantly sparse.

## Materials and methods

This study included 118 in-house patients from Shaukat Khanum Memorial Cancer Hospital and Research Centre, Lahore, Pakistan, with a biopsy-proven diagnosis of squamous cell carcinoma or adenocarcinoma, who received neoadjuvant chemoradiation followed by surgery, and whose five-year follow-up data were available in the hospital archives.

Inclusion criteria

All in-house patients with biopsy diagnoses of squamous cell carcinoma or adenocarcinoma of the esophagus were included in the study. All included patients received chemoradiotherapy prior to surgery with follow-up data of at least five years.

Exclusion criteria

Patients with diagnoses other than squamous cell carcinoma or adenocarcinoma on esophageal resection were excluded from the study. Esophagectomy without prior diagnosis, esophagectomy done at any other hospital, patients who underwent upfront surgery, who were lost to follow-up, or whose five-year follow-up data was not available were excluded from the study.

A retrospective analysis of 118 cases was performed for the period 2013 to 2018. The study was approved by the Institutional Review Board (IRB) of Shaukat Khanum Memorial Cancer Hospital and Research Centre (IRB number: EX-02-02-24-04, dated March 8, 2024). Pretherapy clinical staging was tabulated for all patients. Data on the current status of all patients, together with any site of progression of disease after surgery, were retrieved from the archives. Slides for each case were prepared by entirely submitting the gross tumor bed. A histopathological review of esophagectomy slides was carried out by two histopathologists. Factors that were assessed included pathological ypT, ypN status, presence or absence of lymphovascular and perineural invasion, margin status, and chemotherapy response score (CRS) as per College of American Pathologists (CAP) guidelines. Cases were stratified into one of the four categories: complete response (CRS score 0 - chemotherapy response noted with no viable cancer cells); near complete response (CRS score 1 - chemotherapy response present with single cells or rare small groups of cancer cells); partial response (CRS score 2 - chemotherapy response present with residual cancer showing evident regression but more than single or rare groups of cells); and poor or no response (CRS score 3 - no chemotherapy response and no evident tumor regression).

Other findings noted during the histopathological review included the therapy-induced changes of inflammation, fibrosis, foreign body giant cell reaction, necrosis, and the margin status. Overall survival (OS) was calculated from the time of diagnosis to the time of death from any cause. Progression-free survival (PFS) was calculated from the time of diagnosis to the time of relapse, recurrence, or metastasis after surgery.

Preneoadjuvant lymph nodes were clinically and radiologically assessed, and data was extracted from hospital archives. Downstaging was assessed on histological evaluation of lymph nodes. Most lymph nodes showed no chemotherapy changes, and a few showed fibrosis or lymphohistiocytic inflammation. Downstaging was labeled when clinically or radiographically positive lymph nodes were found to be negative on histological evaluation after chemotherapy.

Data was entered and analyzed in IBM SPSS Statistics for Windows, Version 29 (Released 2021; IBM Corp., Armonk, New York, United States). Continuous variables were expressed as median (range). For the descriptive statistics of categorical variables, frequency and percentages were used, and for the comparison of categorical variables, the chi-square test of independence was used. Kaplan-Meier survival analysis was conducted to compare the survival distribution by CRS for both the OS and PFS. The log-rank test was used to test the equality of survival distributions. Cox regression analysis was performed for prognostic variables. A p-value ≤ 0.05 was considered significant.

## Results

At the time of data analysis, 68 patients (57.6%) had died. The median OS of all patients was 65 months. The five-year survival rate was 59.6%. On univariate analysis, the following variables were identified to be prognostic factors to determine the OS: age, clinical T stage (cT), pathological T stage (ypT0), pathological N stage (ypN), and CRS. Gender, histological grade, and clinical N stage (cN) did not affect OS. The ypT stage and CRSs were further categorized, and each category was a significant prognostic factor in univariate analysis. This is illustrated in Table 1.

**Table 1:** Demographic features of patients with univariate analysis of clinicopathological features n represents the number of patients A p-value is considered statistically significant when p < 0.05

Characteristics	Number of patients, n (%)	Hazard ratio	95% confidence interval	p-value
Age, median (range)	51 (26-76)	-	-	0.076
Gender				0.488
Male	62 (52.5)	1.185	0.734-1.913	
Female	56 (47.5)	1		
Site of tumor				0.023
Mid	10 (8.5)	1		
Lower	81 (68.6)	2.997	1.026-8.757	0.045
Gastroesophageal junction	27 (22.9)	1.567	0.562-4.371	0.390
Histology				0.000
Adenocarcinoma	15 (12.7)	1		
Squamous cell carcinoma	103 (87.3)	3.611	1.942-6.714	
Histological grade				0.271
Well	19 (16.1)	1		
Moderate	81 (68.6)	2.035	0.856-4.837	0.108
Poor	18 (15.3)	1.438	0.705-2.936	0.318
Clinical T stage				0.033
cT2	5 (4.2)	1		
cT3	93 (78.8)	0.962	0.200-4.633	0.962
cT4	20 (16.9)	2.536	1.156-5.562	0.020
Clinical N stage				0.441
cN0	34 (28.8)	1		
cN1	75 (63.6)	0.547	0.216-1.389	0.205
cN2	9 (7.6)	0.670	0.286-1.572	0.358
Clinical M stage				0.152
cM0	116 (98.3)	1		
cM1	2 (1.7)	0.353	0.085-1.469	
Pathological T stage				0.044
ypT0	78 (66.1)	1		
ypT1	6 (5.1)	0.445	0.243-0.850	0.014
ypT2	17 (14.4)	0.243	0.055-1.081	0.063
ypT3	17 (14.4)	0.711	0.318-1.588	0.405
Pathological N stage				0.000
ypN0	94 (79.1)	1		
ypN1	16 (13.6)	0.028	0.003-0.255	0.001
ypN2	7 (5.9)	0.042	0.004-0.402	0.006
ypN3	1 (0.8)	0.105	0.011-1.048	0.055
Chemotherapy response score (CRS)				0.000
0	80 (67.8)	1		
1	14 (11.9)	0.178	0.091-0.348	0.000
2	12 (10.2)	0.112	0.039-0.324	0.000
3	12 (10.2)	0.284	0.115-0.704	0.007

**Figure 1:**
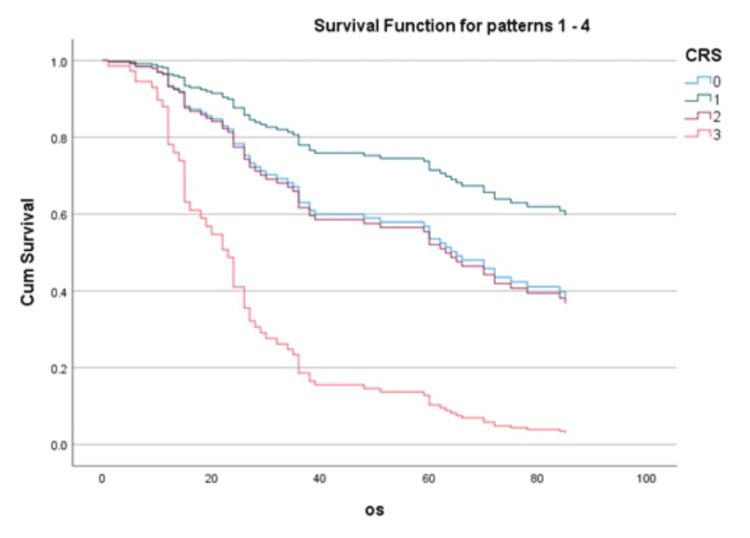
Overall survival curves at various chemotherapy response scores (in months) OS: overall survival; CRS: chemotherapy response score

The median OS for ypT0 was significantly longer (72 months ± 9.701) compared to that for ypT2 (36 months ± 16.464) and ypT3 (24 months ± 8.918). Similarly, the median OS for CRS 0 was significantly prolonged (72 months ± 9.714) compared to CRS 2 (32 months ± 5.196) and CRS 3 (13 months ± 1.732). This concludes that advancing the ypT stage and CRS score had a worse prognosis. OS curves at various CRS scores are shown in Figure 1.

At the time of data analysis, during a follow-up period of at least six years, 37 patients (31.35%) out of 118 had disease progression, either in the form of recurrence at an anastomotic site (seven patients, 5.9%) or metastasis to the brain (six patients, 5.08%), mediastinal lymph nodes (three patients, 2.5%), cervical lymph nodes (four patients, 3.3%), retroperitoneal lymph nodes (two patients, 1.6%), pleura (three patients, 2.5%), lung (11 patients, 9.32%), liver (five patients, 9.3%), and adrenal gland (one patient, 0.08%). The median PFS for all ypT stages and CRS scores was 82.933 ± 4.488 months. Like the OS, PFS decreased significantly with the increase in ypT stage and CRS scores. This is illustrated in Table 2.

**Table 2:** Progression-free survival for various ypT stages and CRS scores CRS: chemotherapy response score; PFS: progression-free survival in months (mean value)

CRS score	PFS
CRS 0	97.35
CRS 1	91.64
CRS 2	37.33
CRS 3	15.86
ypT stage	
ypT0	95.04
ypT1	99.00
ypT2	69.72
ypT3	29.47

Multivariate analysis using the Cox proportional hazard model for survival is illustrated in Table 3. Age, sex, site, histological grade, cT, cN, cM, ypT, ypN, and CRS were entered into the model. On multivariate analysis, only CRS score 2 was found statistically significant, as shown in Table 3.

**Table 3:** Multivariate analysis for survival outcomes A p-value is considered significant when p < 0.05

	Hazard ratio	95% confidence interval	p-value
Age	1.005	0.980-1.031	0.689
Gender			0.950
Male	1.019	0.566-1.833	
Female (reference)	1		
ypT0	1		0.834
ypT1	0.994	0.212-4.650	0.994
ypT2	0.641	0.139-2.951	0.568
ypT3	1.265	0.416-3.847	0.678
ypN0	1		0.304
ypN1	0.000	0.000-1400.215	0.282
ypN2	0.000	0.000-1495.187	0.285
ypN3	0.000	0.000-4053.411	0.344
CRS 0	1		0.278
CRS 1	0.381	0.064-2.254	0.287
CRS 2	0.292	0.078-1.091	0.067
CRS 3	0.328	0.079-1.358	0.124

## Discussion

In our study, out of the 118 specimens, 14 (11%) were adenocarcinoma, one (0.8%) was adenosquamous carcinoma, and the remaining 103 (87%) were squamous cell carcinomas, thereby supporting the fact that esophageal squamous cell carcinoma is more prevalent in Asian populations compared to adenocarcinoma [9]. Out of 14 adenocarcinomas, 12 (85%) had died at the time of analysis, with 11 (78%) patients showing disease progression compared to 55 deaths (53%) and 25 disease progressions (24%) of squamous cell carcinoma. This is supported by the work of Mariette et al., whereby adenocarcinoma had shorter OS [10]. In this study, one patient with adenosquamous histology died after 10 months.

There are various models to stratify therapy response, and their utility has been studied in different studies [11]. However, our study incorporated only the CAP protocols of CRS for assessing the therapy response.

This study shows that chemoradiation-treated esophagectomy specimens show a trend in improved outcomes with increasing response toward therapy. A direct positive relation can be established between pathological stage status or CRS status with survival. Age, ypT, ypN, and all CRS scores were statistically significant in univariate analysis. This aligns with the work of Rohatgi et al., Chirieac et al., and Kitasaki et al. [12-14]. However, only CRS score 2 was statistically significant in multivariate analysis in our study. This is supported by various other studies [13,15]. This is also supported by the work of Davarzani et al. [16], whereby non-responders (tumor regression grade 4-5) had higher mortality risk compared to responders (tumor regression grade 1-3), which is in contrast to the study of Kitasaki et al. [14].

In our group of patients, 69.5% had downstaged primary tumors, 55.08% had downstaged lymph nodes, and 67.7% had pathological complete responses (CRS score 0). This proportion was much higher than in many other studies [1,12-13,17-18].

The five-year OS rate of pretreated patients was 59.6%. This is comparable to the five-year OS (52.6%) in the work of Liao et al. [5] and significantly greater than that observed in various other studies [15,19].

The pre-therapeutic or clinical stage did not affect the survival, re-emphasizing the fact that long-term prognosis depends on the response to neoadjuvant chemotherapy.

The significance of ypTNM as an individual prognostic factor has been described variably in different studies. In our study, none of ypT, ypN, and ypTNM could be established as independent prognostic factors on multivariate analysis. This is in line with the work of Hagens et al., whereby complete regression in the lymph nodes was associated with improved survival, yet this failed to reach statistical significance [20]. This is in contrast to various other studies [3,14,16,21-22] where ypN was useful in multivariate analysis. One of the reasons could be the non-linear relation of ypT and ypN. Response in the primary tumor does not always parallel the response in the lymph node. However, similar to the study by Donohoe et al. [23], 66% of our completely regressed tumor (CRS score 0) cases also had ypN0 compared to 61.5%. This emphasized the need for developing systems to incorporate downstaged lymph nodes impacting the survival outcomes [9,16,19,22-24] for partially or incompletely regressed tumors (CRS score 1, 2, and 3). This is also supported by the work of Zanoni et al., in which downstaged lymph nodes had worse survival than natural N0 lymph nodes (lymph nodes with no metastasis) [24].

Even in the downstaged lymph nodes in our study, therapy response changes were not significantly evident. The work of Nieman et al. showed that therapy response changes in the lymph nodes were associated with worse survival. This emphasized that the current practice of counting lymph nodes with no viable tumor but showing tumor necrosis as negative nodes result in understaging [19]. The work of Tsekrekos et al. shows that a combination of the Becker tumor regression grading system with a three-tiered grading of regression in lymph nodes generates a system that is reproducible [25]. Also, studies have found that early tumors responded well to therapy as compared to tumors at later stages [6]. This may be attributed to a lower tumor burden in the early stages.

Our study could not establish the importance of metastatic disease overriding the status of the primary tumor, unlike the work of Tong et al. [3].

The resection margin is considered an important factor in the success of surgical management. The work of Dexter et al. showed that a tumor within 1 mm of the resection margin was an independent prognostic factor [26]. In our study, 11% (n = 13) of the cases had residual tumors within 1 mm of the adventitial resection margin, and although this could not be established to be statistically significant, all these cases had early disease progression. Only one case had all margins positive and a CRS score of 3 (highest residual tumor burden) and consequently the lowest PFS and OS.

In our study, lymphovascular and perineural invasion was found in six (5%) and seven (5.9%) cases of those with residual tumors, respectively. Although a statistical relation could not be established due to the smaller numbers, all cases with lymphovascular and perineural invasion had early disease progression. This is supported by the work of Kitasaki et al., whereby lymphovascular invasion was an independent prognostic factor [14].

A lot has been studied to predict the behavior of esophageal squamous cell carcinoma at cellular and molecular levels. The work of Shigeoka et al. has shown that high densities of tumor-associated CD204+ macrophages were associated with poorer disease-free survival [27]. High CD8 infiltration before and after nCRT, as well as CD3 and CD4 infiltration after neoadjuvant chemotherapy, generally correlated with better prognosis. A high expression of tumoral or stromal programmed death-ligand 1 (PD-L1) after neoadjuvant therapy was generally associated with poor prognosis. Moreover, total lesion glycolysis (TLG) and metabolic tumor volume (MTV) of the primary tumor were potentially predictive for clinical prognosis [28].

Studies have shown that immune-related genes (like ABL1, ATF2, CD38, ICOSLG, and many others) were significantly related to patients' OS and that immunohistochemically identified tumor-infiltrating CD4+ and CD8+ T lymphocytes would also help to categorize the low and high risk of unfavorable prognosis [29].

There are a few limitations of our study as well. Firstly, the study sample was limited. Secondly, this study only included the study population from a single center. This emphasizes the need for conducting larger studies with larger cohorts to determine OS and PFS.

## Conclusions

Our study has shown that a positive relationship can be established between improving CRSs and OS/PFS. A similar relationship can be established between decreasing pathological T stage and improved OS/PFS. Further, the percentages of the downstaged primary tumor, downstaged lymph node, complete pathological response, and five-year OS were greater in our study as compared to many previous studies. Only CRS score 2 was found statistically significant on multivariate analysis, and such significance could not be established for ypN status. Our study emphasizes the need for developing grading/scoring systems whereby downstaged lymph nodes could be incorporated into the therapy response assessment.
